# Management of isolated retroperitoneal Castelman’s disease: A case report

**DOI:** 10.1016/j.ijscr.2020.03.048

**Published:** 2020-04-22

**Authors:** Ben Ismail Imen, Hakim Zenaidi, Yahmadi Abdelwahed, Rebii Sabeur, Zoghlami Ayoub

**Affiliations:** Department of General Surgery, Trauma and Burns Center, Ben Arous, Tunisia

**Keywords:** Castelman disease, Surgical management, Retroperitoneal tumor, Unicentric, Angiofollicular lymphoid hyperplasia

## Abstract

**Introduction:**

Castleman’s disease (CD) is a rare disorder characterized by benign angio follicular lymphoid hyperplasia, with gentle behavior. Its etiology and prevalence are unclear. It is usually found in the mediastinal area and it is rare to see this tumor in the retroperitoneum.

**Presentation of case:**

A 53 year old male with a retroperitoneal hypervascular calcified mass densely adherent to the psoas muscle that was accidentally encountered on a CT scan. Provisional diagnosis of retroperitoneal liposarcoma was made and the patient underwent an exploratory laparotomy. The histologic examination of the surgically resected specimen disclosed « a hyaline type of Castleman’s disease ».

**Discussion:**

Castleman’s disease, is a rare heterogenous group of lymphoproliferative disorders that arises mostly in the mediastinum. The retroperitoneal localization is very rare. Clinically, CD can be subdivided into a localized form (unicentric disease) and a generalized form (multicentric disease) according to the number of lymph nodes involved. A careful histological and immunohistochemical examination of the tumor tissue after surgery is the only way for tumor diagnosis.

**Conclusion:**

Castelman’s disease is a rare entity that has often benign and non invasive behavior, but remains a diagnostic challenge. There is no reliable diagnostic method and its definitive diagnosis is based on histopathology report. For treating the unicentric variant, radical surgical resection is considered to be the gold standard.

## Highlights

• The unicentric form of Castelman's disease (CD) is rarely present as an isolated retroperitoneal tumor.
• Because of its rarity even experienced surgeons may misdiagnose retroperitoneal CD.
• Surgeons should consider this disease because surgical resection is the only treatment of the unicentric form.

## 1 Introduction

Castleman’s disease (CD) is a rare disorder characterized by benign angio follicular lymphoid hyperplasia first described by Castleman et al. in 1956 [1]. Because of the rarity of this disease, epidemiological data are not available. The etiology of CD is still poorly understood, and is sometimes associated with human immuno-deficiency virus (HIV) and human herpes virus 8 (HHV-8) [2].

The most common location of CD is mediastinum (70%) but the involvement of extrathoracic sites like neck, axilla and pelvis have also been reported. CD in the retroperitoneum is especially unusual, accounting for only 7% of all reported cases (400 patients so far) [3]. Castleman’s disease has been classified as unicentric (localized) form and multicentric (systemic) form based on clinical and radiological findings [4]. Histopathologically, the disease is divided into the hyaline vascular, plasma cell or mixed cell type [5]. Diagnosis can be suggested by preoperative morphologic imaging (ultrasonography with Doppler, computed tomography scan, magnetic resonance imaging and angiography) but definitive diagnosis can only be obtained with surgical pathology [6]. Optimal therapy for unicentric disease is surgical resection and is curative if lesion is completely resected. Multicentric disease is primarily treated with systemic therapies.

We herein present a rare case of unifocal retroperitoneal mass proved to be Castleman’s disease.

This work has been reported in line with the SCARE criteria [7].

## 2 Case presentation

**Fig. 1:**
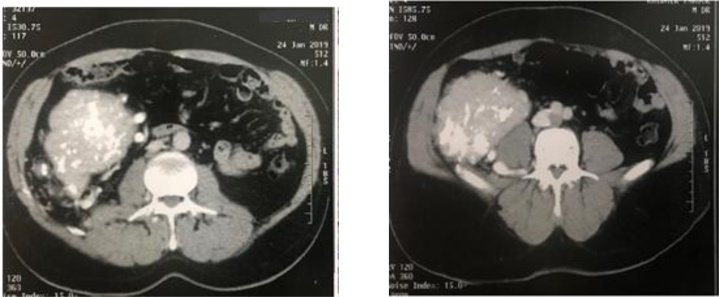
CT scan of the abdomen shows a well-defined enhancing mass.

A 53-year-old male, with a history of a squamous cell carcinoma of the lower lip was admitted to our surgical department for an asymptomatic retroperitoneal mass. The lesion was incidentally discovered on a thoraco-abdomino and pelvic CT scan done as part of the follow-up of the squamous cell carcinoma of the lower lip. No abnormal clinical findings were recorded, notably no palpable mass. Routine blood investigations including hematological and biochemical tests were normal. Serum alpha-fetoprotein (AFP), CEA and CA 19-9 were unremarkable. The Contrast enhanced computed tomography (CT) of the abdomen and pelvis showed a homogenous enhancing mass lesion in the retroperitoneum, with lobulated contours measuring 26 × 88 × 150 mm, massively calcified with significant collateral venous circulation (Fig. 1).

Based on the radiological findings, a provisional diagnosis of atypical retroperitoneal liposarcoma was made and the patient was prepared for surgical resection. The mass being calcified and hypervascular, it was considered any form of pre-operative biopsy (even fine needle) might be difficult and carry a high risk of bleeding.

**Fig. 2:**
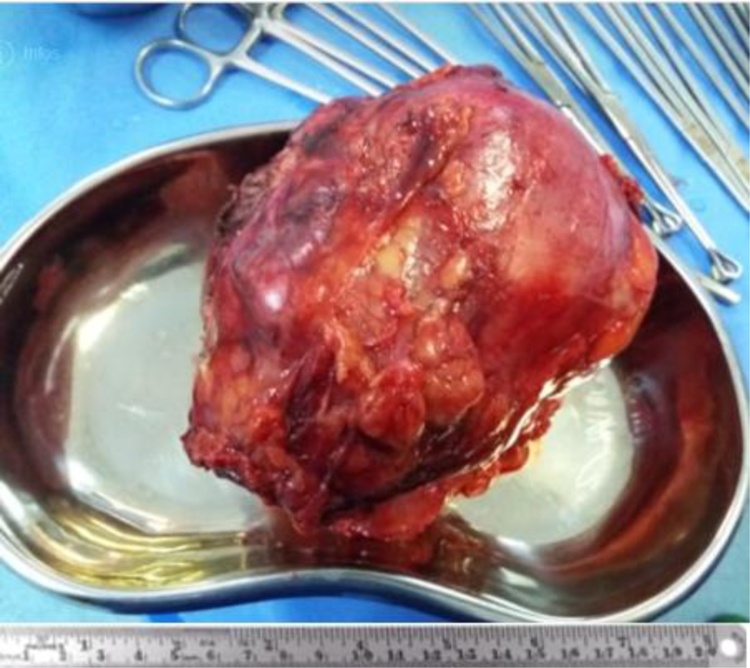
Macroscopic view of the specimen after resection.

At laparotomy, a retroperitoneal large mass of size 15 cm × 8 cm was found. The tumor was limited posteriorly by the psoas muscle, forward by the caecum and laterally by the parietal muscles. The retroperitoneal mass was widely dissected and completely excised along with its capsule, carefully and without complications (Fig. 2).

**Fig. 3:**
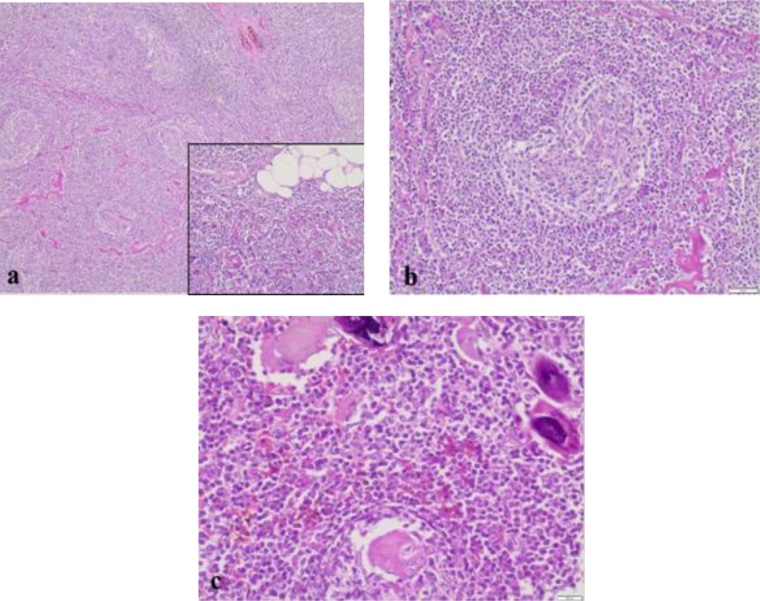
Histological examination: Castleman’s disease of hyaline vascular type. (a) Lymphoid tumor in soft tissue (adipose tissue visible at the level of the cartridge image). (HE, Low magnification). (b) Atrophic follicles of the Castleman type vascular hyaline. (HE, medium magnification). (c) Presence of ossification foci within the lesion. (HE, medium magnification).

**Fig. 4:**
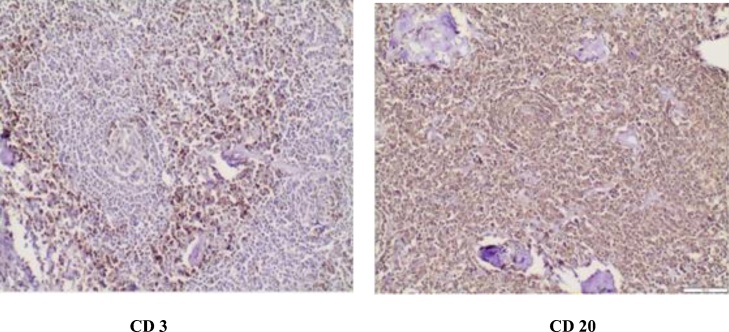
The immunohistochemical staining revealing positive findings of CD3 and CD20.

The microscopic examination revealed an enlarged lymphnode composed of multiple follicles of various sizes with involuted germinal centers (Fig. 3). The immunohistochemical study revealed CD20 (+, follicles), CD3 (+, parafollicles) and Bc12 (+, follicles) (Fig. 4). These findings were consistent with Castleman’s disease hyaline vascular variant.

The patient had an uneventful postoperative course and was discharged on the fourth postoperative day. He is now nine months post surgery and is well and without any evidence of disease. He is being followed-up at six monthly intervals.

## 3 Discussion

Castleman’s disease, also known as angio follicular lymphnode hyperplasia, is a rare heterogenous group of lympho-proliferative disorders. It was first reported by Benjamin Castleman in 1956 [1]. CD is a kind of rare pathology, usually benign, and its incidence is estimated at 21–25 cases per million person-years [8].

The pathogenesis of CD is not clearly defined. However, an increased production of IL-6 by lymphnodes appears to have a pivotal role in the development of the disease [9]. CD is more common in young adults without predilection of sex, although age varies from 8 to 66 years [5].

Castlman’s disease arises mostly in the mediastinum (70%) neck, abdomen, axilla, shoulder, orbit, pelvis, pancreas, leptomeninges, vulvar, and retroperitoneum have also been reported as locations of CD [10]. Retroperitoneal localization is very rare and has been reported to account for 7% [3].

Clinically, CD can be subdivided into a localized form (unicentric disease) and a generalized form (multicentric disease) according to the number of lymphnodes involved [4]. The Clinical manifestations have great differences between the two forms. UCD is generally asymptomatic and may be diagnosed incidentally on imaging [11].

Multicentric disease commonly occurs in the setting of HIV infection along with HHV 8 infection and it affects multiple lymphnode regions [2]. The patients often present with systemic symptoms such as fever, night sweats, general malaise, weight loss, and anemia. Generalized lymphadenopathy and hepatosplenomegaly are commonly seen in these patients [12].

According to the histological pattern, CD may be classified into several subtypes based on its specific features, including hyaline vascular variant, plasma cell variant, plasmablastic variant, and mixed cell subtype [13].

As reported in the literature the hyaline vascularsub type is the most common one, accounting for approximately 90% and it presents as a unicentric disease in 76%–90% of cases, while the plasma cell variant commonly presents as multicentric disease in 80%–90% of cases [14].

The preoperative diagnosis of CD remains a great challenge especially in case of CD located in the retroperitoneal space. Imaging tests have been shown to aid diagnosis. On ultrasonography, CD is seen as a homogeneously hypoechoic mass. In contrast-enhanced CT, CD manifests as homogeneous or heterogeneous mass of soft-tissue density with rim enhancement and slow washout. In magnetic resonance imaging, lesions are heterogeneous with increased signal on T2 and T1 [15]. Although, MRI is superior to CT as it shows better soft tissue delineation, but is also not definitive for the diagnosis of CD. Of course, since no study with a high sample size has been conducted in this field, sensitivity and specificity of none of these diagnostic methods were exactly identified [11].

Ultimately, a careful histological and immunohistochemical examination of the tumor tissue after surgery is the only way for tumor (and its type) diagnosis [11]. Therefore, preoperative diagnosis is often not achievable. In fact, a preoperative needle biopsy or fine needle aspiration is not recommended because of the difficulty of achieving an adequate amount of tissue, the possibility of spreading tumor cells and the risk of severe bleeding in hypervascular mass [6]. Similar to most of the cases reviewed in the litterature, a surgical resection was used for the diagnosis and treatment of the present patient.

The clinical subtypes of Castleman’s disease are associated with different prognoses and treatments. Unicentric Castleman’s disease has a good prognosis (no recurrence and 5-year survival rate of nearly 100%) and is treated by the radical surgical resection [16].

For the systemic form, no curative therapies have been found yet. Treatment options in MCD include immunotherapy with monoclonal antibodies directed at IL-6 (siltuximab), antiviral, antiretroviral agents, and chemotherapeutic agents (doxorubicin, vincristine, cyclophosphamide, melphalan, and chlorambucil) [15]. Multifocal Castleman’s disease, has a poor prognosis and may progress to lymphoma (5%) and hence, regular surveillance is necessary for these patients [17].

With the advent of laparoscopic surgery, laparoscopic resection has been selected in recent reports. Jhen-Hao et al. have demonstrated, in a systematic review of the litterature, that the laparoscopic approach is feasible for retroperitoneal CD and it leads to better results than open surgery as it reduces postoperative pain and limits the length of hospital stay [2]. The mean size of the laparoscoply resected masses was 5 cm with extremes ranging from 2 to 7.5 cm. The tumors were placed in a plastic bag and removed through the umbilical trocar site or a Pfannensteil incision [2]. To the best of our knowledge, there has not been a reported case of laparoscopic resection of a large calcified CD over 10 cm in diameter. In cases of an uncertain nature mass, the laparoscopic approach must be considered as the last diagnostic tool and the first treatment one.

## 4 Conclusions

Castelman’s disease is a rare entity that has often benign and non invasive behavior, but remains a diagnostic challenge. There is no reliable diagnostic method and its definitive diagnosis is based on histopathology report. Although rare it should be included in the differential diagnosis of retroperitoneal mass. For treating the unicentric variant, radical surgical resection is considered to be the gold standard.

## Declaration of Competing Interest

Authors declare no conflict of interest.

## Sources of funding

This study was not supported by any institution and company.

## Ethical approval

Ethical approval was not required and patient identifying knowlage was not presented in the report.

## Consent

Written informed consent was obtained from the patient for publication of this case report and accompanying images. A copy of the written consent is available for review by the Editor-in-Chief of this journal on request.

## Author contribution

Study concepts: Dr Zenaidi Hakim.

Manuscript writing: Dr Ben Ismail Imen.

Helped in data interpretation and manuscript evaluation: Dr Rebii Saber.

Data acquisition: Dr Yahmadi Abdelwahed.

Critical revision: Dr Zoghlami Ayoub.

## Registration of research studies

NA.

## Guarantor

Dr Ayoub Zoghlami.

## Provenance and peer review

Not commissioned, externally peer-reviewed.

## References

[1] Castleman B., Iverson L., Menendez V.P. (1956). Localized mediastinal lymphnode hyperplasia resembling thymoma. Cancer.

[2] Jhan J.H., Li C.C., Wu W.J., Lee H.Y. (2018). Isolated retroperitoneal Castleman’s disease: a case report and literature review. Clin. Case Rep..

[3] Aygun C., Tekin M.I., Demirhan B., Peskircioglu C.L., Agildere M., Ozkardes H. (2000). A case of incidentally detected Castleman’s disease with retroperitoneal paravertebral localization. Int. J. Urol..

[4] Casper C. (2005). The aetiology and management of Castleman disease at 50 years: translating pathophysiology to patient care. Br. J. Haematol..

[5] Keller A.R., Hochholzer L., Castleman B. (1972). Hyaline-vascular and plasma-cell types of giant lymphnode hyperplasia of the mediastinum and other locations. Cancer.

[6] Bracale U., Pacelli F., Milone M., Bracale U.M., Sodo M., Merola G. (2017). Laparoscopic treatment of abdominal unicentric Castleman’s disease: a case report and literature review. BMC Surg..

[7] Agha R.A., Borrelli M.R., Farwana R., Koshy K., Fowler A., Orgill D.P., For the SCARE Group (2018). The SCARE 2018 statement: updating consensus surgical CAse REport (SCARE) guidelines. Int. J. Surg..

[8] Munshi N., Mehra M., van de Velde H., Desai A., Potluri R., Vermeulen J. (2015). Use of a claims database to characterize and estimate the incidence rate for Castleman disease. Leuk. Lymphoma.

[9] Ravet M.B., Peuchmaur M., Devergne O., Audouin J., Raphael M. (1991). Interleukin-6 gene expression in Castleman’s disease. Blood.

[10] Bucher P., Chassot G., Zufferey G., Ris F., Huber O., Morel P. (2005). Surgical management of abdominal and retroperitoneal Castleman’s disease. World J. Surg. Oncol..

[11] Rajabiani A., Abdollahi A., Farahani Z. (2015). Asymptomatic isolated retroperitoneal Castleman’s disease: a case report. Iran. J. Med. Sci..

[12] Jaafreh A.A., Alaqqad A., Alqasem K., Daghmin A.A. (2017). Unicentric retroperitoneal Castleman disease presenting with pleural effusion. Anat. Physiol..

[13] Vries I.A., van Acht M.M., Demeyere T., Lybeert M.L., de Zoete J.P., Nieuwenhuijzen G.A. (2010). Neoadjuvant radiotherapy of primary irresectable unicentric Castleman’s disease: a case report and review of the literature. Radiat. Oncol..

[14] Jindal S., Adithya K., Madaan V., Gupta R., Vohra1 S., Tandon V., Govil D.A. (2018). Rare cause of retroperitoneal mass: Castleman’s disease. Apollo Med..

[15] Gopi P., Potty V.S., Kaurav R.S., Govindan K. (2018). Unicentric Castleman’s disease as a localized retroperitoneal mass: a case report and review of literature. Int. J. Appl. Basic Med. Res..

[16] Chronowski G.M., Ha C.S., Wilder R.B., Cabanillas F., Manning J., Cox J.D. (2001). Treatment of unicentric and multicentric Castleman disease and the role of radiotherapy. Cancer.

[17] El‑Osta H.E., Kurzrock R. (2011). Castleman’s disease: from basic mechanisms to molecular therapeutics. Oncologist.

